# Targeting autophagy to treat HIV immune dysfunction

**DOI:** 10.1080/27694127.2023.2254615

**Published:** 2023-09-11

**Authors:** Wenli Mu, Heather Martin, Anjie Zhen

**Affiliations:** a Division of Hematology/Oncology, Department of Medicine, David Geffen School of Medicine at UCLA, Los Angeles, CA, USA; b UCLA AIDS Institute and the Eli and Edythe Broad Center of Regenerative Medicine and Stem Cell Research, David Geffen School of Medicine at UCLA, Los Angeles, CA, USA

**Keywords:** Ant-HIV-1 immunity, autophagy, HIV-1 immunopathogenesis, IFN-I signaling, inflammation, rapamycin, immune exhaustion

## Abstract

Chronic immune activation and inflammation are hallmarks of Human Immunodeficiency Virus-1 (HIV-1) pathogenesis. Therefore, approaches to safely reduce systematic inflammation are essential to improve immune responses and thus slow or prevent HIV progression. Autophagy is a cellular mechanism for the disposal of damaged organelles and elimination of intracellular pathogens. It is not only vital for energy homeostasis, but also plays a critical role in regulating immunity. However, how it regulates inflammation and antiviral T cell responses during HIV infection is unclear. Our study demonstrated that impairment of autophagy leads to spontaneous type I-Interferons (IFN-I) signaling, while autophagy induction reduces IFN-I signaling in macrophages. Importantly, we demonstrated that in vivo treatment of autophagy inducer rapamycin in chronically HIV infected humanized mice decreased chronic IFN-I signaling, improved exhausted anti-viral T cell function, and reduced viral loads. Taken together, our study supports the therapeutic potential of rapamycin and potentially other autophagy inducers in alleviating HIV-1 immunopathogenesis and improving anti-viral T cell responses.

## Abbreviations

HIV, Human Immunodeficiency Virus-1; CTL, cytotoxic T lymphocytes; ART, anti-retroviral therapy; IFN-I, Type I interferons; IFNAR1, IFN-I receptor; PBMC- peripheral blood mononuclear cells; MX1, MX dynamin like GTPase 1; IRF7, Interferon Regulatory Factor 7; ISGs, Interferon-Stimulated Genes; ATG5, autophagy-related gene 5

Human Immunodeficiency Virus-1 (HIV-1)-specific CD8+ cytotoxic T lymphocytes (CTLs) are crucial in controlling HIV-1 replication and eliminating HIV-1-infected cells. However, due to immune evasion by HIV-1 and the development of dysfunctional HIV-1-specific T cells, natural CTLs fail to durably control HIV-1 replication in the absence of anti-retroviral therapy (ART). Driven by chronic immune activation, T cell exhaustion remains one major barrier to achieve sustained immune surveillance of HIV-1 infection. Type I interferons (IFN-I) are critical for viral control during acute infections. However, mounting evidence indicates that chronic IFN-I signaling can lead to T cell exhaustion during chronic HIV-1 infection. Previously, we and others have demonstrated that treating humanized mice with IFNAR1 (IFN-I receptor) blockers after established HIV-1 infection, functionally rescues HIV-1-specific T cells, decreases hyperimmune activation, and reduces the size of HIV-1 viral reservoirs in combination with ART. However, as IFN-Is are key immune regulators, more specific approaches to curb inflammation are necessary.

Autophagy is a homeostatic mechanism involved in the disposal of damaged organelles, protein aggregates, and elimination of intracellular pathogens. Modulation of the autophagy process is important for controlling microbial pathogen infections and importantly, the induction of autophagy inhibits HIV-1 release. Autophagy is also critical for T cell homeostasis, survival, and memory formation. Interestingly, peripheral blood mononuclear cells (PBMCs) from HIV-1-controllers display higher levels of autophagy activity than normal progressors, suggesting that autophagy plays an important role in limiting viral pathogenesis.

Intriguingly, recent discoveries suggest that autophagy and IFN-I signaling are tightly linked. Our recent study found that induction of autophagy could fine tune IFN-I responses [1]. In the monocytic THP1 cell line, autophagy impairment by ATG5 (autophagy-related gene 5) knockdown led to spontaneous upregulation of IFNβ and the Interferon-Stimulated Genes (ISGs) MX dynamin like GTPase 1(MX1) and Interferon Regulatory Factor 7(IRF7). We also found that autophagy inducers rapamycin and spermidine, which activate autophagy via distinct pathways, downregulated ISGs expression in stimulated primary macrophages from healthy donors. Additionally, we demonstrated that the downregulation of ISGs by rapamycin and spermidine in THP1 cells is dependent on ATG5, suggesting that the downregulation of IFN-I responses by rapamycin or spermidine, is mediated by autophagy [1].

Rapamycin, an mTOR (mammalian target of rapamycin) inhibitor, is a well-characterized autophagy inducer. Importantly, it is an FDA-approved drug for the prevention of transplant rejection and has an excellent safety profile, including in HIV-1-positive patients. In HIV-1 infected humanized mice, treatment of the animals with low doses of rapamycin reduced viral load and ISGs expression in multiple lymphoid tissues, suggesting a regulation of the IFN-I immune responses by autophagy [1]. In addition, our study showed that combinational treatment with ART and rapamycin of HIV-1 infected humanized mice effectively induced autophagy levels, decreased T cell activation and exhaustion markers, and reduced chronic elevated IFN-I signaling to levels similar to uninfected animals. Importantly, we found that rapamycin treatment alone reduced viral load. Combined treatment of rapamycin and ART led to accelerated viral suppression and significantly lowered viral rebound after ART withdrawal, approximately 10 fold. The rapamycin treated mice also harbored less HIV-1 DNA and RNA in the blood and spleen.

We investigated how rapamycin impacted HIV-1-specific CTLs responses using ex vivo cytokine assays. CTLs from infected control mice produced significantly lower levels of pro-inflammatory cytokines compared to healthy controls after PMA/Ionomycin stimulation, suggesting functional exhaustion of T cells in HIV-1-infected mice. In contrast, T cells from infected mice treated with rapamycin produced increased levels of pro-inflammatory cytokines in response to both PMA/ionomycin and HIV specific stimulation using a HIV-1 peptide pool as compared to controls. Taken together, our data show that rapamycin treatment significantly improved cytokine production of CTLs in response to mitogen or viral peptide stimulation, suggesting improved T cell functions, which could in turn lead to better viral control.

In summary, our data revealed important regulatory cross talk between autophagy and IFN-I responses. Furthermore, we demonstrated that autophagy inducer rapamycin treatment reduces chronic IFN-I signaling and improves CD8+ T cell function in HIV-1 infected humanized mice, resulting in lower viral rebound. Our study provided important insights into autophagy’s regulation of IFN-I signaling and T cell function. It also indicates the therapeutic potential of autophagy inducers to treat HIV-1 mediated hyperinflammation and T cell dysfunction. We anticipate that the IFN-I modulatory effects by autophagy inducers, observed in this study and others, could have applications beyond the treatment of HIV-1 infection, especially for other chronic diseases that involve persistent inflammation.
